# Ethical, legal, and social issues of AI use in emergency healthcare: a scoping review

**DOI:** 10.1186/s12911-026-03355-x

**Published:** 2026-03-13

**Authors:** James Edgar Lim, Fahad Javaid Siddiqui, Angela Ballantyne, Michael Dunn, Sinead Prince, Dominic Wilkinson, Jonathan Lewis, Sungwon Yoon, Julian Savulescu, G. Owen Schaefer

**Affiliations:** 1 Centre for Biomedical Ethics, Yong Loo Lin School of Medicine, National University of Singapore, 10 Medical Dr, #02-03 MD 11, Singapore, 117597 Singapore; 2 Pre-Hospital & Emergency Research Centre, Duke-NUS Medical School, 8 College Rd, Singapore, 169857 Singapore; 3 Department of Primary Health Care and General Practice, University of Otago, PO Box 7343, Wellington, Otago New Zealand; 4 Uehiro Oxford Institute, 16-17 Saint Ebbe’s St, Oxford, OX1 1PT United Kingdom; 5 School of Law, Faculty of Arts, Humanities, and Social Sciences, Ulster University, York St, Belfast, Belfast, BT15 1ED UK; 6 Health Services Research and Population Health, Duke-NUS Medical School, 8 College Rd, Singapore, 169857 Singapore

**Keywords:** Artificial intelligence, Emergency healthcare, Ethics, Scoping review, AI ethics

## Abstract

**Background:**

Advances in artificial intelligence (AI) systems suggest that they can be used to improve healthcare outcomes via diagnosis, prognostication, patient management, risk assessment, etc. AI systems could be particularly useful in emergency healthcare (EHC) by synthesizing data to generate accurate conclusions rapidly. But the use of AI in EHC raises ethical, legal, and social concerns.

**Objective:**

The present study undertakes a scoping review to collate, map, and synthesize existing literature on the ethical, legal and social issues (ELSIs) associated with AI in EHC. The aim was to assess which ELSI issues were recognized and analyzed in the current literature and which were under-explored.

**Design/Methods:**

Online databases were used to identify papers published on the identified topic. An initial search strategy of IEEE, Pubmed, and Scopus yielded 156 unique records; 40 records underwent textual review, after which another 7 were excluded due to scope. The final 33 were analysed for content.

**Results:**

Overall, the literature was mostly positive towards AI applications on EHC, with key themes aligning with the general AI ethics literature: transparency, bias, benefit/harm, justice, accountability, privacy and trust. Analyses of these issues, however, were mostly superficial and did not substantially engage with some of the distinctive features of EHC like urgency and high-stakes decision-making. In particular, urgency and stakes were under-recognised or under-explored in the EHC AI literature. Arguably, urgency in some emergency scenarios could justify more flexible ethical/regulatory standards, while conversely high-stakes contexts might require more stringent standards.

**Conclusion:**

Lack of discussion of these contextual nuances suggests a significant gap in the literature of deeper research into the unique ethical, legal and social issues arising from AI use in EHC. This paper extends current knowledge by highlighting the need for deeper and more contextualized investigation of AI ethics in EHC.

**Trial registration:**

Not applicable.

**Supplementary Information:**

The online version contains supplementary material available at 10.1186/s12911-026-03355-x.

## Introduction

Emergency healthcare (EHC) stands out as one area of healthcare where the use of artificial intelligence (AI) can make a significant contribution. In health emergencies, medical practitioners make life-or-death decisions with limited time, resources, and information. AI systems can assist practitioners in making decisions by gathering data and synthesizing it to generate conclusions (like diagnoses) rapidly, with relatively minimal resource demands, and perhaps more accurately than practitioners themselves. As a result, AI systems, at least in principle, may improve clinical outcomes while contributing to more effective and cost-efficient delivery of EH services.

At the same time, the use of AI in EHC raises ethical, legal, and social issues (ELSIs) like the need for explicability, interpretability, transparency, or interpretability [1–5], the degree of bias [3, 4, 6, 8, 33], issues around patient consent or autonomy [9, 10, 33], accountability for decisions made [3, 6, 11], and respect for privacy [5, 9, 13, 14, 34], While many ELSIs related to EHC represent more general concerns with the use of AI, there are some unique issues that arise because of the urgency associated with EHC. For instance, there are particular challenges in ensuring accountability when medical practitioners do not have the time to engage in shared decision-making with patients [15]. 

Examples of uses of AI systems in EHC settings include triage [16], abnormality detection in emergency radiology [1], prediction of conditions like sepsis in intensive care [17], supporting nursing workflow [34], risk stratification [18], prediction of neurological recovery after cardiac arrest [19], and in prehospital emergency care [20]. 

To our knowledge, no attempts – including systematic and scoping reviews – have been made to map out the ELSIs related to the use of AI in emergency care specifically. In response, this scoping review aims to collate, map, and synthesize existing literature on the ELSIs associated with the use of AI in EHC, with respect to the research question:

What are the ethical, legal, and social issues (ELSI) related to the use of AI for decision-making in emergency medicine?

This scoping review will identify key themes, gaps in knowledge, and areas requiring further investigation. This will in turn, help researchers focus their efforts on productive agendas, as well as inform policy guidance.

## Methodology

This scoping review uses Arksey and O’Malley’s [21] methodological framework for scoping reviews in determining the extent and scope of current research within our topic, and identifying noteworthy gaps in the research. This framework describes an “iterative” and “reflexive” process that identifies five key steps: [1] identifying a research question; [2] identifying relevant studies; [3] study selection; [4] charting the data; and [5] collating, summarizing, and reporting the results [21]. It complies with PRISMA guidelines for scoping reviews [22]. The protocol can be provided on request from the corresponding author of this article.

### Search strategy

Only papers published in English were considered for this scoping review. A team member, in consultation with a librarian, identified a list of search terms. The key terms were “Ethics”, “Legal”, “Social” (while initially included, “social” was later excluded because it resulted cluttering due to too many irrelevant results retrieved), “Concerns”, “implications”, “artificial intelligence”, “aided decision making”, and “emergency and intensive care settings”. Additionally, the terms “prehospital,” “ambulance,” and “paramedic” were also included to ensure we included prehospital care. Studies relevant for our scoping review were identified by searching IEEE, Scopus, and Pubmed/Medline (Initially, results from Philpapers were also considered. However, Philpapers did not yield any identifiable records using the search terms). Grey literature was not included in this review. No limits on the study design or year of publication were applied. The final search strategy for Pubmed can be found in Additional File 1: Lit Search Implementation Steps.

### Study selection

A record was included at the title/abstract stage if (a) it discussed some application of AI or other algorithmic decision-making tools to emergency healthcare (including triage, emergency department, pre-hospital emergency services, and intensive care), *and* (b) if it discussed any ethical, legal, or social issues. Records that discussed ELSIs related to intensive care, but did not mention EHC were included during the screening phase. This is because of some overlap between intensive care and emergency healthcare cases (that is, some intensive care cases are also emergency cases, or involve dimensions of urgency), and overlap in many ELSIs related to the two types of cases. For example, allocation of scarce resources in intensive care requires the use of triage processes and principles – many of which will be similar in both EHC and intensive care. So that we did not prematurely exclude relevant discussions, we included records which discussed intensive care.

At the full-text screening stage, the record should also discuss (c) ELSIs relevant to the use of the AI in EHC. Records which only discussed ELSIs which were *not* relevant to the application of AI to EHC were excluded. Records which discussed non-AI decision-making tools were also excluded. Two members of the team (JEL and OS) screened the records at the title/abstract stage to determine relevance, and made decisions to include or exclude based on consensus. At the full-text screening stage, two other members of the team independently reviewed each text, flagging records that did not meet the above criteria to the team leader. The team leader and one other member of the team (JEL and OS) made final decisions regarding inclusion and exclusion of papers according to the criteria based on a synthesis of the reports by the two members of the team.

### Charting the data

The methodologies chosen for this review were thematic and numeric analysis (which involved counting the total number of papers which discussed a given theme). Thematic analysis “consists of examining excerpts of text and asking how this text relates to the research question” [23]. To this end, we recorded information according to three main categories. First, the use cases discussed in the record (e.g. the use of AI to assign triage scores to patients). We included both existing use cases studied in a paper, or hypothetical use cases suggested or explored in the paper. Second, the normative themes discussed in a paper. Following Braun and Clarke [24], we developed a set of normative themes using a reflexive method. Two members of the team (JEL and OS) conducted a preliminary reading of a subset of the total number of records, using observations in this reading to generate an initial list of codes (an initial list of interesting ideas which could be organized into themes) [24]. These codes were reviewed and consolidated into a list of core ethical, legal, and social issues (as indicated in the research question), which we labelled as the normative themes. The themes were reviewed and refined as members of the research team read and catalogued their observations on the normative issues raised in each paper. Third, we extracted information about the basic details of empirical investigations (if any), mapping the forms of investigation, target groups, location of study, sample sizes, and primary conclusions. Fourth, we noted the subjective “overall impression” each paper had of the use of AI in EHC – whether a paper seemed to present a “mostly positive” impression, a “mostly negative” impression, a mostly neutral impression, or did not seem to have an overall impression.

The team leader developed a data extraction tool for researchers to record the relevant information from the papers using Google Forms. The extraction form was pilot tested and calibrated based on a smaller subset of all the papers, by two members of the team. Each paper was independently analysed by two team members, and the data were compiled by the data extraction tool and downloaded into an Excel spreadsheet. Results (from the two team members for each paper) were screened by the team leader for discrepancies. When any discrepancies were noted, the team leader compared the two accounts with each other and the paper to synthesize a final account which best matched the accounts and the paper.

## Results

**Fig. 1:**
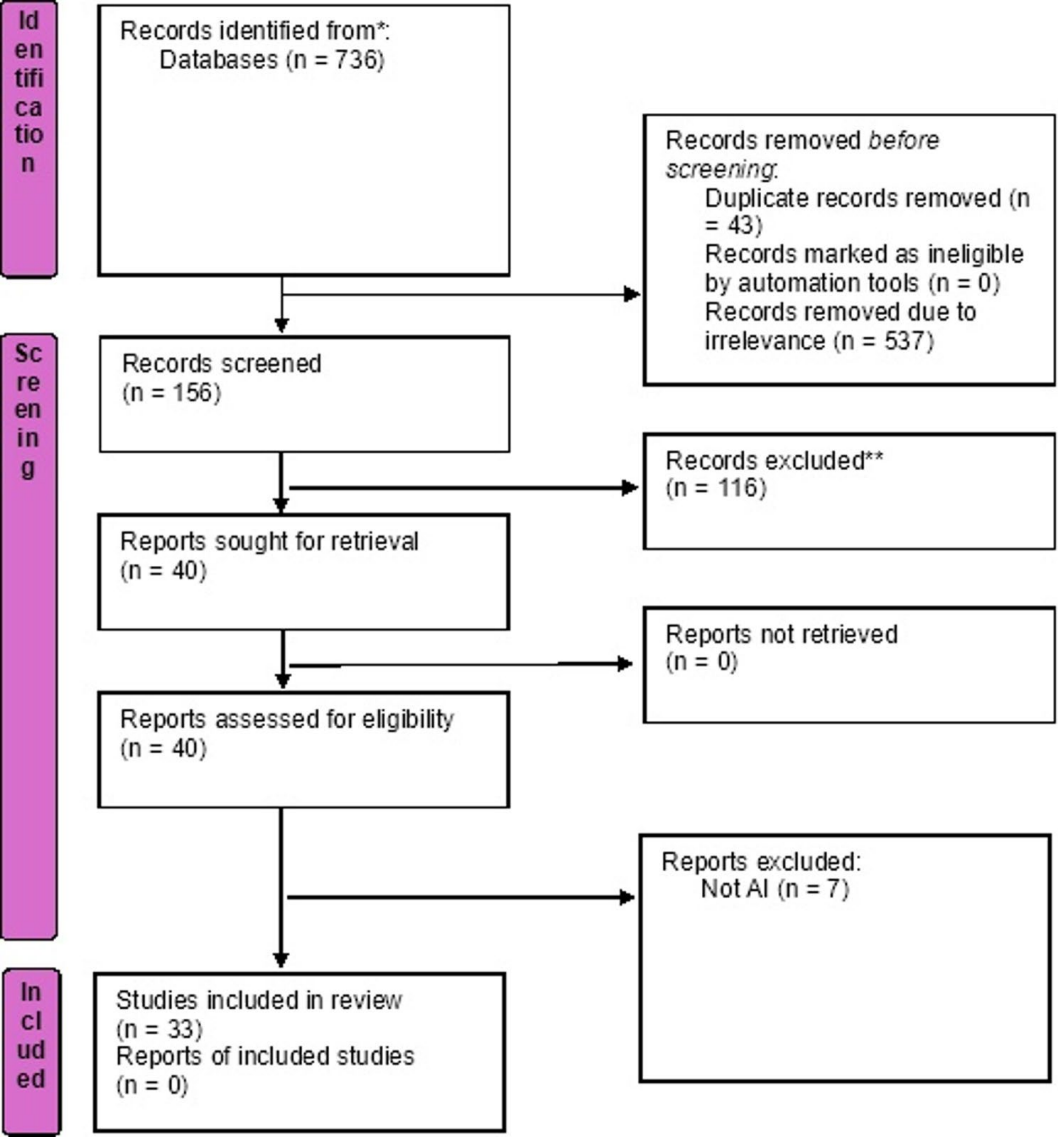
PRISMA flowchart

In the first broader search strategy, 736 results from IEEE were retrieved, 956 from Pubmed, and almost 4000 records from Scopus. Consequently, the second search strategy was added with ‘emergency medical services’ (EMS) and related terms ensuring relevance and manageability of the retrieved literature. This search strategy yielded 199 results from IEEE, Pubmed, and Scopus. After screening for duplicates, 156 unique records were obtained. The resulting list of records was compiled in Excel. After title/abstract screening, forty records were deemed eligible for full-text screening and analysis. Following full-text screening, 33 records were deemed eligible for analysis We have indicated this process in Fig. 1.

### Charting the data

Of the 33 papers included in this study for data extraction, the vast majority (32/33) were published within a ten-year period from this study (2015–2025). This is unsurprising because of the very recent advancements in AI capabilities. A summary of data items and key results in the 33 papers are in Table 1.

**Table 1:** Summary of data items and key results

S/*N*	Author(s) & Year	Type of Study/Analysis	Use Cases	Key Normative Themes
1	Agrawal et al. (2023)	Online survey of Healthcare professionals (emergency radiologists), United States	Triage within radiology, diagnostic in emergency radiology, prediction/ prognostication	Accuracy, bias, explainability/interpretability, trust, human-in-the-loop, accountability, privacy/security
2	Ahmed & Alsisi (2022)	Bayesian probability analysis	Triage, ICU admission prioritization, prediction and risk stratification	Benefits and risks, bias, explainability/interpretability, consent, trust, intuition vs. algorithm, regulation, justice/equity, stress reduction for clinicians
3	Al-Anazi et al. (2024)	Narrative review	Diagnostic (respiratory care), monitoring, prediction, treatment decision support	Benefits and risks, bias, explainability/interpretability, consent, trust, empathy, moral accountability, justice/equity, privacy/security, governance, regulation
4	Alexandropoulou et al. (2023)	Systematic review	Diagnosis & prediction of sepsis in ICU	Benefits and risks, bias, explainability/interpretability, autonomy, trust, accountability, justice, regulation, privacy
5	Alkhachroum et al. (2020)	Literature review	Triage, prediction in neurocritical care, monitoring	Benefits and risks, bias, explainability/interpretability, trust
6	Almagharbeh (2024)	Survey of Healthcare professionals (nurses), Jordan	Prediction and risk stratification, AI-based DSS (decision support systems) in nursing workflow, monitoring	Efficiency/accuracy vs. errors, explainability/interpretability, relevance of personal intuition, empathy
7	Barea Mendoza et al. (2024)	Narrative review	Risk prediction, early detection and monitoring, clinical management, admin support	Benefits and risks, bias, explainability, autonomy, trust, justice/equity, regulation
8	Beil et al. (2019)	Ethical analysis	Prognostication in intensive care	Benefits/risks, bias, explainability, consent/autonomy, trust, empathy, moral/legal accountability, justice/equity/fairness, regulation, privacy law, governance
9	Bienefeld et al. (2024)	Systematic task analysis, survey, interview of policy makers and doctors, International panel	Monitoring, documentation, data analysis, prescribing medication and treatment, diagnosis, patient interaction, prediction and risk stratification	Bnefits/risks, bias, explainability, relevance of personal judgment, empathy/human touch, moral/legal accountability, equity, regulation
10	Biesheuvel et al. (2024)	Narrative review	Triage, diagnosis, prediction	Benefits/risks, bias (generalisability), explainability/interpretability, trust, human judgement/intuition, moral accountability, regulation, privacy/security
11	Bignami et al. (2023)	Review	Diagnosis of COVID-19, peri-operative management, monitoring in ICU, prediction	Benefits/risks, transparency/explainability, bias, consent/autonomy, privacy/security, governance
12	Bishara et al. (2022)	Theoretical review	Diagnosis, prediction	Benefits/risks, bias, interpretability, legal accountability, justice/equity, privacy/security, governance, regulations
13	Boverhof et al. (2024)	Systematic review	Triage, diagnosis, stroke care workflow, data abstraction in EHC, discharge decisions from ICU, and the optimization of discharge timing	Benefits/risks, bias, explainability, consent/autonomy, trust, security, moral/legal accountability, regulation, privacy law, privacy/security, governance, IP/knowledge sharing
14	Denecke & Baudoin (2022)	Narrative review	Triage, diagnosis, prediction, monitoring, home care robots	Benefits/risks, bias, explainability, trust, moral/legal accountability, privacy law, privacy/security, governance
15	Feretzakis et al. (2024)	Retrospective model analysis	ML for ED triage	Benefits/risks, explainability, trust, privacy/security, moral accountability, justice/equity
16	Gheysen & Rex (2023)	Literature review	Diagnostics, monitoring (anesthesiology), prediction and risk stratification, education/training, pain monitoring	Benefits and risks, transparency/explainability, bias, relevance of personal judgement, moral/legal accountability
17	Gorges & Ansermino (2020)	Narrative review	Diagnostics (pneumonia/sepsis), prediction, monitoring, airway management	Benefits and risks, Explainability/interpretability, trust, consent/autonomy, regulation
18	Haley et al. (2024)	Qualitative survey of parents of paediatric patients, United States	Diagnosis, prediction, treatment decision support	Benefits and risks, bias, explainability, autonomy/consent, privacy/security, justice/equity
19	Lysaght et al. (2024)	Ethical analysis	Prognostication/prediction in ICU	Benefits/risks, bias, explainability/interpretability, autonomy/consent, trust, relevance of intuition, empathy, moral/legal accountability, justice, privacy/confidentiality
20	Makwana et al. (2022)	Clinical AI tool development	Prediction of neurological recovery after cardiac arrest	Benefits/risks, Bias, explainability/interpretability, privacy law, justice/fairness, regulations
21	Montomoli et al. (2024)	Ethical analysis	General clinical decision support systems	Benefits/risks, bias, explainability/transparency, moral accountability, justice/equity, regulation, governance, privacy
22	Neves et al. (2020)	Ethical analysis	Use of AI for triage/resource allocation during pandemic	Explainability/transparency, trust, justice/equity, governance
23	Nord-Bronzyk et al. (2025)	Ethical analysis	Triage tools, risk assessment in implementation	Benefits/risks, explainability/interpretability, trust, autonomy/consent, empathy
24	Pammi et al. (2023)	Narrative review	Multiomics and AI for prediction and monitoring in perinatology; early diagnosis, risk prediction, monitoring	Benefits/ risks, Bias, Explainability/interpretability, autonomy, privacy/security, fairness/justice, legal accountability
25	Petersson et al. (2023)	Qualitative study/interview of healthcare professionals, Sweden	Mortality prediction with AI for ED admit/discharge decisions (triage), diagnostics	Benefits/risks, bias, explainability/transparency, autonomy/consent, trust, legal accountability, justice/equity, regulation, governance
26	Pinsky et al. (2024)	Ethical Analysis	Diagnostics, prediction of patient deterioration, monitoring and life support	Benefits/risks, bias, explainability/interpretability, consent/autonomy, trust, relevance of personal judgment, moral accountability, privacy law, privacy/security, regulations, justice/ equity, governance, IP issues and knowledge sharing
27	Rostam Niakan Kalhori (2022)	Technical review	Triage improvement, diagnosis of chest pain, risk stratification, monitoring	Potential benefits and complications
28	Shearer & Baruch (2022)	Ethical analysis	Use of decision aids and AI in ED, diagnostic tools, risk stratification	Empathy/human touch, importance of personal intuition/judgement
29	Stewart et al. (2024)	Qualitative study/interviews of healthcare professionals (EHC consultants), Australia	Triage, diagnostics, discharge prediction, monitoring	Benefits/risks, bias, explainability/interpretability, trust, relevance of personal intuition, legal accountability, justice/equity, privacy
30	Townsend et al. (2023)	Qualitative study/interviews of healthcare professionals (EHC practitioners), United Kingdom	Triage, diagnostics	Benefits/risks, explainability/interpretability, trust, relevance of personal intuition, empathy, moral/legal accountability, regulation, privacy/security, governance
31	Vinay et al. (2021)	Narrative Review	ICU triage	Benefits/risks, bias
32	Vitt & Mainali (2024)	Narrative review	Triage, Diagnosis, prediction, monitoring	Benefits/risks, Bias, explainability/interpretability, justice/fairness, privacy/security, privacy law
33	Yang et al. (2004)	Narrative review	IT-based decision-support for neonatal intensive care (not AI-specific), prediction and risk stratification	Empathy/human touch

The majority of the papers (23/33) were best characterized either as reviews (of the literature, policy, and/or existing capabilities), as theoretical pieces focused on explicating ethical or social issues, or both. 7 papers involved some empirical methods – surveys, qualitative studies, structured interviews, mixed methods, etc. – with the goal of determining the views of stakeholders. Three studies involved the use of model development or statistical analysis to determine or prove the effectiveness of some AI or algorithmic tool. The key themes found in empirical studies are summarized in Table 2.

**Table 2:** Detailed summary of key themes in empirical studies

Study	Method	Population	Location
Agrawal, A., et al. (2023)	Survey	Healthcare professionals (emergency radiologists)	United States
Almagharbeh, W. T. (2024)	Survey	Healthcare professionals (nurses)	Jordan
Bienefeld, N., et al. (2024)	Task analysis, survey, interview	Policy makers and doctors	International
Haley, L. C, et al., (2024)	Survey	Parents of paediatric patients	United States
Petersson, L., et al., (2023)	Interviews	Healthcare professionals	Sweden
Stewart, J., et al. (2024)	Interviews	Healthcare professionals (EHC consultants)	Australia
Townsend, B. A., et al. (2023)	Interviews	Healthcare professionals (EHC practitioners)	United Kingdom

We catalogued the use cases discussed by the papers, which we categorized in the following way in Table 3.

**Table 3:** Frequency of use cases discussed (the number of use cases exceeds the number of records because many papers discussed more than one use case)

Use Case	*n*
Triage	15
Diagnostics	20
Prediction & Risk Stratification (for non-triage cases)	25
Monitoring	14
Prediction of Discharge from ED	1
Decision-making Support (e.g. offering solutions)	2
Data Extraction & Analysis (e.g. trend identification)	3
Training/Education for Healthcare Workers	1
Care Robots/Patient Interaction	2
Clinical Management/Admin Support/ Documentation	2
Prescribing Medication/Treatment	1

Based on subjective overall impressions, over half of the papers seemed mostly positive about the introduction of AI in emergency care (19/33). Smaller numbers either did not present an overall conclusion (8/33) or were mixed (6/33). No papers were overall negative about the use of AI in EHC. While there was some subjectivity in determining the overall impression of a paper, there was agreement among reviewers that no paper was overall negative.

Finally, we catalogued the normative themes discussed in the records. These normative themes represent the ethical, legal, and social issues discussed in the literature. The results are represented in the following Table 4.

**Table 4:** List of normative themes catalogued by in this study

Normative Theme	Explanation	No/33
Transparency, Explainability, Interpretability, or Explicability	Concerns regarding opaque/black-box AI, and whether it is important for practitioners or other parties to understand how AI models work.	29
Potential Benefits and Risks of Harm	How AI can affect patient outcomes, including discussions about accuracy, resource use, speed, wrongful diagnosis, deskilling or overdependence on AI.	29
Bias	Whether AI will be biased or more/less effective for certain demographics.	24
Trust	Whether AI can be trusted; whether the use of AI might undermine trust in medical practitioners and institutions; whether systems which employ AI can be trusted; and the effects of lack of trust.	19
Justice/Equity/Fairness	Whether the benefits and burdens of AI use will be fairly distributed.	18
Privacy/Security	Whether the use of AI risks violating anyone’s *moral* rights to privacy.	17
Moral Accountability	Who ought to take moral responsibility for AI decisions.	13
Patient Consent/Autonomy	Whether the use of AI might violate patient autonomy, or whether informed consent is required for the use of AI.	15
Regulations	What regulations are required for the justified use of AI in EHC?	15
Legal Accountability/liability	Who is liable for mistakes made by AI decisions	13
Governance	Which regulatory bodies should be responsible for the governance of AI in healthcare, and how should they approach the issue?	11
Importance of Empathy in Medicine	Whether the use of AI will render medical treatment less empathetic.	10
Relevance of Human Intuition/Personal Judgment	Concerns about the role human judgment plays in medical decisions, and whether humans should be kept “in the loop” for these reasons.	8
Privacy law	Whether the use of AI violates anyone’s *legal* privacy rights.	7
Intellectual Property (IP) Issues and Knowledge Sharing	The ethical implications of companies maintaining IP rights over AI systems, and whether they ought to share AI systems.	2

The above table only represents the frequency of each normative issue discussed. In this study, we did not seek to categorize or quantify each paper according to how extensively it discussed an issue. Nevertheless, we note that in many of the papers, normative issues were discussed in cursory or superficial ways, either because the paper had other primary aims or because the paper was a review of multiple issues.

### Collating, summarizing, and reporting the results

The most commonly raised normative issues were: Transparency, Potential Benefits and Risks of Harm, Bias, Trust, Justice, and Privacy. Each of these issues were discussed in about half the papers or more. In the following paragraphs we provide a qualitative overview of the discussion surrounding these core concepts in the papers reviewed.

### Transparency, explainability, interpretability, and explicability

Transparency, explainability, interpretability, and explicability are closely related concepts that together received the most frequent mention in our results (29/33). In technical papers, these terms are not interchangeable, and are used to refer to different properties of an AI system [25]. “Interpretable AI” refers to AI models which are inherently transparent [26]. “Explainable AI” refers to AI models for which we have secondary post-hoc models which tell us how the primary model works [27]. “Explicability” refers to a combination of “intelligibility” (as an answer to the question “how does it work?”) and in the ethical sense of “accountability” (as an answer to the question: “who is responsible for the way it works?”)” [28]. In contrast, we found that within the papers analysed in this study, authors did not clearly distinguish transparency, explainability, interpretability, or explicability, and instead used the terms in reference to a more central normative concern: whether practitioners and patients can have a sufficient understanding of how decisions are made [2, 29, 30]. 

Among the key reasons cited for why transparency/ explainability/ interpretability/ explainability are important is the perceived need to secure the trust of users. Multiple authors either noted or argued that practitioners and patients are more likely to trust AI systems that they can understand [3, 4, 15, 29–33]. These observations are corroborated by some of the empirical records, which note that many participants view some form of explainability or transparency as crucial for trust [1, 11]. 

Another set of reasons for the importance of transparency/ explainability/ interpretability/ explainability are moral and legal accountability. Understanding how or why an AI system made its decisions is important so that we can scrutinize those decisions and assign responsibility accordingly [2, 31, 33]. 

### Potential benefits and risks of harm.

The other most raised issue (29/33) related to potential benefits and risks of harm. We have catalogued some of the most prominent potential benefits and risks of harm in the following Table 5.

**Table 5:** List of key potential benefits and risks of harm (items being on the same row do not imply a relationship between them)

Potential Benefits	Risks of Harm
Higher/more reliable precision and/or accuracy in diagnosis and prognosis. [2, 13, 33, 34, 36, 38]	Incorrect diagnoses or recommendations made by AI. [14, 37]
More individualized treatment methods. [38]	Vulnerability to adversarial attacks. [38]
Better patient monitoring, including in hospital or remotely. [5, 38]	Excessive dependence on AI systems. [15, 36]
More accurate or earlier predictions for risk. [36, 38, 39]	Risks associated with a lack of generalizability in deployment across different settings or a lack of generalizability of training data, e.g. risks of using an inappropriate model for a population. [6, 14, 30]
Administrative benefits, including time management in emergency departments, improved documentation, and better clinical management, etc. [34, 36]	Tools which are not easy to use may inhibit hinder adoption. [31]
Streamlining healthcare practices, resulting in reduced costs for patients. [3]	Work overload of healthcare professionals due to complexity or too much data. [31, 40]

On balance, authors seemed to be optimistic about the potential benefits, while also cautiously optimistic that the risks of harm could be mitigated.

*Bias*.

Many papers in this study were concerned with the risk of bias, arising from biased or insufficiently generalizable datasets (24/33), with several papers discussing how the use of AI systems can increase existing disparities for vulnerable groups [3, 5, 17, 30, 33, 36, 38, 39, 41]. This can include bias against racial, ethnic, or cultural minorities [4–6, 15, 17, 42], bias based on gender [6, 15, 17, 43], age [6], sexuality [6], and existing comorbidities [15, 43], At the same time, one empirical study reported that clinicians were less troubled by the possibility of algorithmic bias, and “more about standardization and failing to see each patient as a unique human being, that is, that the recommendation is plausible given a specific patient situation in the clinical context” [2]. 

### Trust in AI/institutions

Finally, several of the papers also discussed the issue of trust in either the AI systems themselves or the institutions that use them (19/33). The theme of trust was relevant to both practitioners and patients, and multiple papers reflected on the importance of trust for either or both groups [4, 5, 11, 41]. While trust was not explicitly defined in the papers, multiple papers indicated that trust was of some instrumental value. In these papers, trust was described as a necessary condition for effective adoption and use of AI systems [11, 13]. The issue of trust was also frequently linked to the issue of explainability, transparency, interpretability, or explicability – several papers claimed that some form of transparency (or explainability, etc.) was necessary for medical practitioners and patients to trust the AI systems [3, 13, 14, 17, 29, 31–33, 37, 38]. Another set of features linked to trust is the way practitioners and institutions implement the use of AI in healthcare, with emphasis on clear communication between practitioners and patients [31, 43]. Lysaght et al. [33] argued that accountability is important for trust formation, writing that “accountable processes in App use with allow for patients and families to trust that their interests are being properly assessed and that decisions are based on sound considerations”. Agrawal et al. [1] also noted that trust in AI systems will depend on the extent of expert agreement on how effective those AI systems are.

### Justice/equity/fairness

Another ELSI discussed by a number of papers was the distribution of benefits and burdens from the integration of AI systems (18/33). This issue is closely related to the issue of algorithmic bias, with some worrying that “in situations where AI systems are trained on biased data sets, the propagation of prejudices is possible, culminating in inequitable or discriminatory outcomes” [38]. However, some worries were distinct. For instance, Bishara et al. [6] claim that those who contribute data to train models should benefit from its deployment, implying a principle that the distribution of benefits should be reciprocal for those who contribute. Another paper expressed worries that AI technologies would not be available to all, with less access for low-income and rural populations [42]. Yet other papers discussed the issue of distributive justice from a different angle: the issue of whether and how AI systems could be used to help allocate resources fairly [3, 30, 32].

### Privacy/security

Several papers also expressed concerns about privacy or data security for users and patients (17/33). For the most part, the papers agreed that it was important to design and deploy AI systems in a way that protected patient privacy [6, 9, 13, 14, 29–31, 38, 43–45]. Empirical studies concurred on this point, with participants in those studies agreeing that privacy was a central concern [5, 11, 34, 42]. At the same time, some papers recognized that data privacy could sometimes come at the expense of other important outcomes (like model accuracy), and that a balance needed to be struck between privacy and other values [13, 15]. 

## Discussion

Our results are overall consistent with findings from similar studies that review the ethical and social issues pertaining to the use of AI in (non-emergency) healthcare, to the extent that they discuss similar ELSIs. Other scoping reviews identified privacy and security [46–49], trust [48], accountability and responsibility [47–50], bias [47, 48], explicability/ transparency/ explainability/ interpretability [46, 47, 49, 50], autonomy or consent [10, 46, 47], prevention of harm and patient safety [46, 49, 50], fairness [49], impacts on the patient-physician relationship [50], and governance [50]. 

Some ELSIs received less attention. These include the relevance of human intuition/personal judgment in medicine, the role of empathy, and IP issues and knowledge sharing. One possible reason for this is that the time-dependent nature of emergency care could mean that improving clinical outcomes with greater accuracy and maximal utility should take greater priority. Thus, if an AI system can improve clinical outcomes with greater efficiency, this may be more important than other values insofar as an emergency department can more quickly and cost-effectively treat more patients. However, neglecting these issues in emergency healthcare may be an oversight, and further studies focused on these issues and their implications for emergency healthcare could constitute novel and valuable contributions to the literature.

This observation is part of a more general insight: we found that in many cases, discussions on the ELSI within the papers studied were fairly cursory. Many of the papers were general reviews of several issues, including applications, technical challenges, and economic considerations (For example, see [6, 19, 38].) As a result, detailed and critical discussions on the ethics of applying AI in EHC were simply not a focus. A few papers – for example [15, 41, 51], –provided detailed discussions of ethical issues. Nevertheless, these papers generally only aimed to review a range of ethical issues and explicate them. Because of the range of ethical issues explored by these papers, they did not engage in a critical and in-depth analysis of any particular ethical issue. These papers may be understood as written for the “practitioner’s perspective”, as an explanation of multiple considerations, rather than from a “ethicist’s perspective”, which prioritizes critical analysis of the theoretical foundations underlying the issues. One possible outcome of this perspective is the relative optimism towards AI. As we noted earlier, no papers within this study were overall negative about AI. While such optimism may indeed be well founded, it is also important to have more critical analysis of the ELSIs related to the use of AI. Further research (particularly empirical) should be conducted to validate this optimism.

Having noted this, a key finding was that many ELSIs were not discussed in relation to emergency medicine specifically. While it may seem that emergency healthcare is just one application of healthcare more broadly, there may be distinct ethical issues that arise in EHC. This is because of key characteristic features of EHC: (i) urgency-sensitive constraints, (ii) lack of sustained doctor-patient relationships (iii) higher levels of uncertainty, (iv) differences in practitioners, and (v) inability to obtain patient consent. Here, we shall briefly discuss how some ELSIs may involve distinct considerations in the context of EHC, as well as point out other observations we made in relation to those ELSIs. In doing so, we also outline an agenda for future research.

### Transparency/explainability/interpretability

Because of the *urgency-sensitive time constraints* in EHC, there may not be enough time for practitioners to think about the explanations for why an AI system made certain recommendations. As such, they may not be able to engage meaningfully with those explanationss [52]. Thus, what counts as an adequate explanation, or what counts as transparent enough, may be different in emergency settings. For example, in non-emergency settings, practitioners may be interested in the details of how an AI system works – which data points it uses to form a conclusion, how it weighs those data points, whether those data points track a patient’s race or gender, etc. But in emergency settings, practitioners may only be interested in *actionable* information which directly affects how they will act. In some cases, this information may involve data points which explain how the AI system works. In other cases, data points used by the AI system may not be actionable – for example, if a patient’s hair colour is used as a proxy to predict certain outcomes [26]. Emergency practitioners may not be interested in such information, and may even be hindered by too much irrelevant data.

Additionally, few papers made specific recommendations about what we should do if transparent AI tools cannot be developed without significant losses in performance (An exception is Boverhof et al., [31] who state that if interpretability is impossible, then some naturally explainable techniques like regression models should be used). A reason for this may be that it is difficult to critically review both the issues and solutions available in the ethics of using AI in EHC. This leaves a significant gap in the research between ethical analysis and technical or regulatory change. Moving forward, ethical frameworks should include some analysis and recommendation on how much trade-off (if any) we ought to accept in favour of transparency, and vice versa. One possibility that practitioners and institutions should simply avoid using AI if it cannot be transparent and effective at the same time. Another possibility – more likely in our view – is that there is *in theory* some advantages in performance that would be worth sacrificing transparency for.

Similarly, other trade-offs may have to be made between other moral considerations such as: autonomy and paternalism (in EHC), ensuring lack of bias and access (for example, if a highly accurate model has a known bias against a small subpopulation), etc.

Steps should be taken to determine how to make trade-offs between ethical considerations. These steps should incorporate plausible ethical theories as well as considered views held by experts, practitioners, and members of the public. One method for collecting the considered views of the public is to use some discrete choice experiment to determine the relative weights of various moral considerations [53]. The results can then be integrated with moral theory with Collective Reflective Equilibrium in Practice (CREP) [54]: an algorithmic decision-making process which finds conclusions that are most consistent with most ethical principles and public beliefs. In principle, this method can yield actionable and specific frameworks for when one moral consideration may be sacrificed for another. This could take the form of some Prioritized Normative Hierarchy (or Hierarchy of Norms), or an algorithmic decision-making procedure.

### Trust in AI/institutions

The issue of trust and trustworthiness is also worth exploring further in the context of EHC. For one, in EHC there may be a lack of a *sustained doctor-patient relationship*. A patient in EHC (who may not be lucid) may not have the chance to build trust in a paramedic. Furthermore, a patient in EHC may not have any knowledge about the tools or systems used by EHC workers. Finally, the urgency and high stakes in EHC contexts means that patients may have to “trust” simply because they have no choice. As a result, any concept of “trust in AI” must grapple with the possibility of people trusting AI systems under conditions where genuine trust is difficult to build. It is entirely possible that trust and trustworthiness are simply not useful concepts in emergency contexts, where people have no time to build trust. A possible research pathway here is to adopt a bifurcated approach to AI ethics, where researchers attempt to develop “trustworthy AI” for non-emergency contexts, and focus on developing safe, reliable, and fair AI in emergency contexts.

More generally, we observed some ambiguities in how the notion of trust is used. First, few of the papers were explicit in how “trust” or “trustworthy AI” is defined. This is an important issue because there are disagreements among philosophers and AI experts on whether trust should be understood as an interpersonal concept (for authors who hold this view, see [55–58]) or if it should be understood functionally (where AI can be considered trustworthy if it fulfils its functions reliably) (see [59–61]). Those who hold the view that trust should be understood interpersonally tend to believe that AI systems cannot, by themselves, be trustworthy, while those who hold the functionalist view tend to believe that AI can be trustworthy. Given that interpersonal trust may be difficult to build in emergency settings, it could make sense to use “trustworthy AI” in a strictly functional sense when discussing EHC (but trust in the interpersonal sense may still be relevant in non-emergency).

Second, “trust in AI” and “trust in institutions” was sometimes discussed without much distinction between the two. But these are distinct issues – one could trust a medical institution that uses AI, without trusting the AI itself. Third, there is a distinction between trust and trustworthiness. But the two can come apart. Trust is often a good thing, but it may be harmful when directed towards an untrustworthy actor [62]. An analysis that distinguishes between the value of trust and the value of trustworthy AI could clarify existing debates on the importance of trust in AI within emergency medicine (and perhaps medicine more broadly).

### Justice/equity/fairness

Issues of justice may also be distinct in EHC, because of *urgency-sensitive constraints*. There already exist well-established worries about whether the benefits of AI use will be fairly distributed, especially for rural or marginalized communities. In emergency settings, these worries may be exacerbated, for instance, if an AI system uses certain features to deprioritize members of certain groups (for example, if a triage system uses a patient’s travel time to conclude that a patient might be beyond saving). But it is also possible for AI systems to mitigate existing inequalities, perhaps through more efficient distributions of resources. As we mentioned earlier, further work in this field should conduct some analysis on how trade-offs should be made between the risks associated with AI and how AI may be able to create more equitable outcomes.

### Accountability

Both moral and legal accountability were mentioned surprisingly few numbers of times. Nevertheless, they are issues which may involve some very distinct considerations in EHC, and therefore deserve more theoretical attention. The *urgency-sensitive constraints* can exert cognitive/emotional pressure on practitioners, and impose epistemic constraints on practitioners, who do not have time to carefully consider their various courses of action. As a result, practitioners also have to make decisions given *higher levels of uncertainty*. In both ethics and law, we recognize that intense cognitive/emotional pressure and uncertainty can mitigate the accountability of an agent. Thus, AI ethicists need to recognize that the accountability of EHC practitioners can be limited in some cases. Frameworks for ensuring and distributing accountability in emergency settings may therefore be different from non-emergency cases.

Additionally, *practitioners in emergency settings are not necessarily the same* as medical practitioners in non-emergency settings. Paramedics do not have the same kind of training as medical doctors. They have different goals – stabilizing a situation and limiting harm caused by particular symptoms. And they arguably work according to different principles (a paramedic’s job may be focused on *satisficing* rather than *maximizing* outcomes, whereas a doctor’s role may sometimes involve working towards the best outcomes for patients). Given that emergency practitioners have different training and different expectations, it could be unfair to hold them to account using the same standards as we do non-emergency practitioners. Consequently, accountability structures for the use of AI in EHC may also look different from structures for the use of AI in non-emergency healthcare.

### Patient consent and autonomy

In EHC settings, there may be *difficulties in obtaining informed consent* from patients. For one, patients may be incapacitated, or too panicked to make informed decisions. In addition, practitioners may not have the time to explain clearly what they intend to do for a patient, and why they intend to do so. Thus, what counts as respecting a patient’s autonomy is different in emergency settings, where not obtaining informed consent is not necessarily a violation of autonomy. Given the urgency involved in emergency care and the possibility of a patient’s altered mental state, different standards and requirements of informed consent may apply [63]. Therefore, there may be unique challenges that arise in the application of AI tools in emergency healthcare. These are challenges that ethicists should explore.

### Other issues and observations

Finally, we want to cover some of the themes found in the empirical studies. Notably, there are a limited number of empirical studies that studied the concerns held by people regarding the use of AI in emergency healthcare. This means that the current research within this area is dominated by expert opinion rather than data-driven findings. While expert opinion is certainly very important, this does mean that there’s a significant gap in the existing literature. More empirical research to validate the identified ELSIs should be conducted. Furthermore, within the papers surveyed in this study, that the populations studied were most frequently medical practitioners from Western countries. To some extent, this may be an outcome of the limitations of our search, which only included English language literature. Nevertheless, it is important for the English-speaking academic community to constantly engage with non-Western cultures. Thus, there remains some room for empirical studies to be conducted on other populations, such as patients and family members, the general public, and East Asian/Southeast Asian populations.

While the studies determined the ethical, legal, or social concerns held by the studied groups, they do not reveal specific insights about how weighty or important these concerns are. Such insights may be important because AI systems sometimes have to make trade-offs between different desiderata, such as accuracy and interpretability [64]. Further studies could be conducted with the goal of obtaining more information about how people weigh various ethical considerations. Studies built with the controlled variation of key considerations could reveal important insights about this information.

One last point worth making is that we should we aware of use cases *not* mentioned extensively by the reviewed papers, which may represent gaps in the literature. For example, the use of care bots was discussed only twice, which may have caused some bias *against* discussing the importance of empathy in care. Similarly, other overlooked use cases may result in certain ELSIs being systematically overlooked.

### Limitations

This scoping review did not include non-English literature. As a result, the study’s findings may be biased towards views or ethical concerns predominant in English-speaking cultures.

Furthermore, the methods of thematic and numeric analysis involved grouping ideas into themes and counting them. These methods combined are unable to make finer evaluations of how themes and concepts are treated. For example, in counting the number of times “trust” is discussed, we did not take into account whether “trust” was being treated as a normative concept, psychological state, or governance principle.

Finally, it did not attempt to make any assessments of the quality or rigor of arguments within the papers studied. Therefore, the study cannot reach conclusions about the normative weight that the relevant ethical, legal, or social issue should be afforded. For example, many records discussed or mentioned concerns about opaque or black-box AI. Yet, some philosophers have argued that the ethical use of AI does not require explainability [10, 26, 65, 66]. This study does not settle such issues.

## Conclusion

In summary, this scoping review collates and synthesizes the English language literature on the ELSIs related to the use of AI in EHC. While there is a large and growing literature about the ethics of AI in healthcare generally, and an expanding body of research about the use of AI in EHC, there is a relatively small number of papers about the ethics of applying AI in EHC. Overall, the existing records seem to indicate general optimism towards the use of AI. A majority of the records in this study were overall positive about the adoption of AI tools, while no studies were characterized as negative about the adoption of AI. However, this positivity tended to be guarded. Whether or not AI should be used in EHC depends on our ability to design and use AI systems in ways which meet a number of ethical desiderata. In principle, it seems that these desiderata can be met, although sustained work, regulation, and good governance will be required to meet the desiderata.

Our results have revealed a number of significant gaps in the research. In general, there is limited research on the ELSIs related to the use of AI systems in the emergency context. This matters because the ethical issues that arise in emergency contexts may differ from the issues which arise in other health contexts, due to the time constraints and the people carrying out the care (e.g. paramedics). Specifically, further empirical research (in English) could study the views and priorities held by under-researched populations, like prehospital emergency health practitioners, patients and their families, or East Asian/ Southeast Asian populations. These groups represent key stakeholders in the contexts in which some AI systems may be deployed. We have also indicated that there are currently few in-depth studies indicating the relative importance of ethical considerations. Further theoretical research could also be very productive if it explores the nature of EHC and the distinct ethical issues which arise in the application of AI in EHC.

## Supplementary Information

Below is the link to the electronic supplementary material.

Supplementary Material 1

## Data Availability

Data is provided within the manuscript or supplementary information files.

## Abbreviations

AI: Artificial intelligence
CREP: Collective Reflective Equilibrium in Practice
EHC: Emergency Healthcare
EMS: Emergency Medical Services
ELSI/s: Ethical, Legal, and Social Issue/s
