# Supplementary material for: Ethical, legal, and social issues of AI use in emergency healthcare: a scoping review
Source: BMC Med Inform Decis Mak. 2026 Mar 13;26:129. doi: 10.1186/s12911-026-03355-x (PMC13097863; doi:10.1186/s12911-026-03355-x)
Supplement: Supplementary file 1 — Supplementary Material 1 [file 12911_2026_3355_MOESM1_ESM.docx]

**Structured research question**

1. What is the current ethical, legal, and social issues (ELSI) landscape for the use of AI for decision-making in emergency medicine, *broadly construed* (includes emergency dept, ICU, pre-hospital/ambulatory care, etc.)?
2. What are the ethical, legal, and social implications (ELSI) of the use of AI for decision-making in emergency medicine, *broadly construed* (includes emergency dept, ICU, pre-hospital/ambulatory care, etc.)?
3. Does the use of AI for decision-making in emergency medicine provide morally relevant benefits? If so, what are they?
4. How does the use of AI for decision-making in emergency medicine affect stakeholders:

**Key words (from question)**

| **Domain** | **Key words** |
| --- | --- |
| Population/Problem | Ethics, Legal, Social  Concerns, implications |
| Intervention/Exposure | Artificial intelligence |
| Control/Reference/Gold standard | Aided Decision making |
| Outcome | Emergency and intensive care settings |

**Search by Duke-NUS**

**PubMed:**

Date run: [Aug 14, 2024]

| **Term searched** | **MeSH** | | | | **Free text** |
| --- | --- | --- | --- | --- | --- |
| P | Terms | SH* | Major | Explode | Terms |
| Ethical | Ethics, Research  Ethics | No | No | yes | or ethic*.mp. |
| Legal |  | No | No | No | Legal* |
| Social | Excluded (too generic, cluttering) |  |  |  |  |
| Perceptions | None | No | No | No | Perception* or concern* or implicat* or applicat* or ramification* or impact* or benefit* or advantage* |
|  |  |  |  |  |  |
| I | Terms | SH* | Major | Explode | Terms |
| Artificial intelligence | Artificial Intelligence | No |  | yes | exp Artificial Intelligence/ or ("artificial* intelligen*" or "Deep learning" or "Machine learning" or "AI" or "pattern recognition" or "comput* simulation" or "data science" or "decision support*" or "neural network*" or "fuzzy logic" or "fuzzy system*" or "natural language*" or "NLP" or "nearest neighb*" or "kNN" or "decision tree*" or "bayes*" or "random forest*" or "RF" or "Dimensionality reduction" or "Principal component" or "PCA" or "Support vector" or "SVM" or "gradient boost*" or "GBM" or "AdaBoost" or "LightGBM" or "XGBoost" or "CatBoost").ti,kf,ab. |
|  |  |  |  |  |  |
| C | Terms | SH* | Major | Explode | Terms |
| Decision making | Decision Making |  |  |  | Decision Making/ or (Decision*.mp. and (aid* or tool* or support*).ti,ab,kf.) or (Decision*.mp. adj5 treatment*.ti,ab,kf.) or (Decision*.mp. adj5 share*.ti,ab,kf.) or (Decision*.mp. adj5 care.ti,ab,kf.) or (Decision*.mp. adj5 clinical.ti,ab,kf.) or (Decision*.mp. adj5 medical*.ti,ab,kf.) |
|  |  |  |  |  |  |
| O | Terms | SH* | Major | Explode | Terms |
| Emergency Medical Services | Emergency Medical Services | No | No | Yes | Exp Emergency Medical Services/ or ((emergency adj5 ("care system*" or medical service* or "medical care" or helpline or triag*)) or prehospital or "pre-hospital" or ambulance* or paramedic* or EMS).ti,ab,kf. |
| Intensive care | Critical Care | No | No | Yes | Exp Critical Care/ or ("intensive care" or "high dependency care" or "high-dependency care").ti,ab,kf. |
|  |  |  |  |  |  |

* = Subheading

**Filter used:** Clinical trial/Cohort studies/Case-control studies… Link to [filters](https://sites.google.com/a/york.ac.uk/issg-search-filters-resource/home/recently-added-filters)

| **Source** | **Version** | **Syntax** |
| --- | --- | --- |
|  |  |  |

**Syntax:**

(((exp Ethics, Research/ or exp Ethics/ or ethic*.mp.) OR (legal*.mp.)) AND (Perception* or concern* or implicat* or applicat* or ramification* or impact* or benefit* or advantage*).ti,ab,kf.) AND (exp Artificial Intelligence/ or ("artificial* intelligen*" or "Deep learning" or "Machine learning" or "AI" or "pattern recognition" or "comput* simulation" or "data science" or "decision support*" or "neural network*" or "fuzzy logic" or "fuzzy system*" or "natural language*" or "NLP" or "nearest neighb*" or "kNN" or "decision tree*" or "bayes*" or "random forest*" or "RF" or "Dimensionality reduction" or "Principal component" or "PCA" or "Support vector" or "SVM" or "gradient boost*" or "GBM" or "AdaBoost" or "LightGBM" or "XGBoost" or "CatBoost").ti,kf,ab.) AND (Decision Making/ or (Decision*.mp. and (aid* or tool* or support*).ti,ab,kf.) or (Decision*.mp. adj5 treatment*.ti,ab,kf.) or (Decision*.mp. adj5 share*.ti,ab,kf.) or (Decision*.mp. adj5 care.ti,ab,kf.) or (Decision*.mp. adj5 clinical.ti,ab,kf.) or (Decision*.mp. adj5 medical*.ti,ab,kf.)) AND (exp Emergency Medical Services/ OR ((emergency adj5 ("care system*" or medical service* or "medical care" or helpline or triag*)) or prehospital or "pre-hospital" or ambulance* or paramedic* or EMS).ti,ab,kf. or (exp critical care/ or ("intensive care" or "high dependency care" or "high-dependency care").ti,ab,kf.))

**Records retrieved:**

Broader search: 915; Narrower search (EMS): 58

**Limit:** Year 2010 – 2024 (aug)

Broader search: 956; Narrower search (EMS): 55
